# Preliminary study on image characteristics of epidermoid cyst under reflection confocal microscope

**DOI:** 10.1111/srt.13141

**Published:** 2022-02-07

**Authors:** Hongyong Sun, Yan Duan, Ruiya Li

**Affiliations:** ^1^ Department of Dermatology People's Hospital of Inner Mongolia Autonomous Region Hohhot Inner Mongolia

To the Editor:

Recently, in clinical work, we examined some patients with epidermoid cysts with reflective confocal microscopy (skin computed tomography [CT]). By collecting real‐time dynamic skin CT images of these patients, we found that epidermoid cysts have some common characteristics under skin CT. In addition, we also compared the skin CT images with the typical histopathological features of epidermoid cysts, and found that there was a corresponding relationship and consistency between them.

**FIGURE 1:**
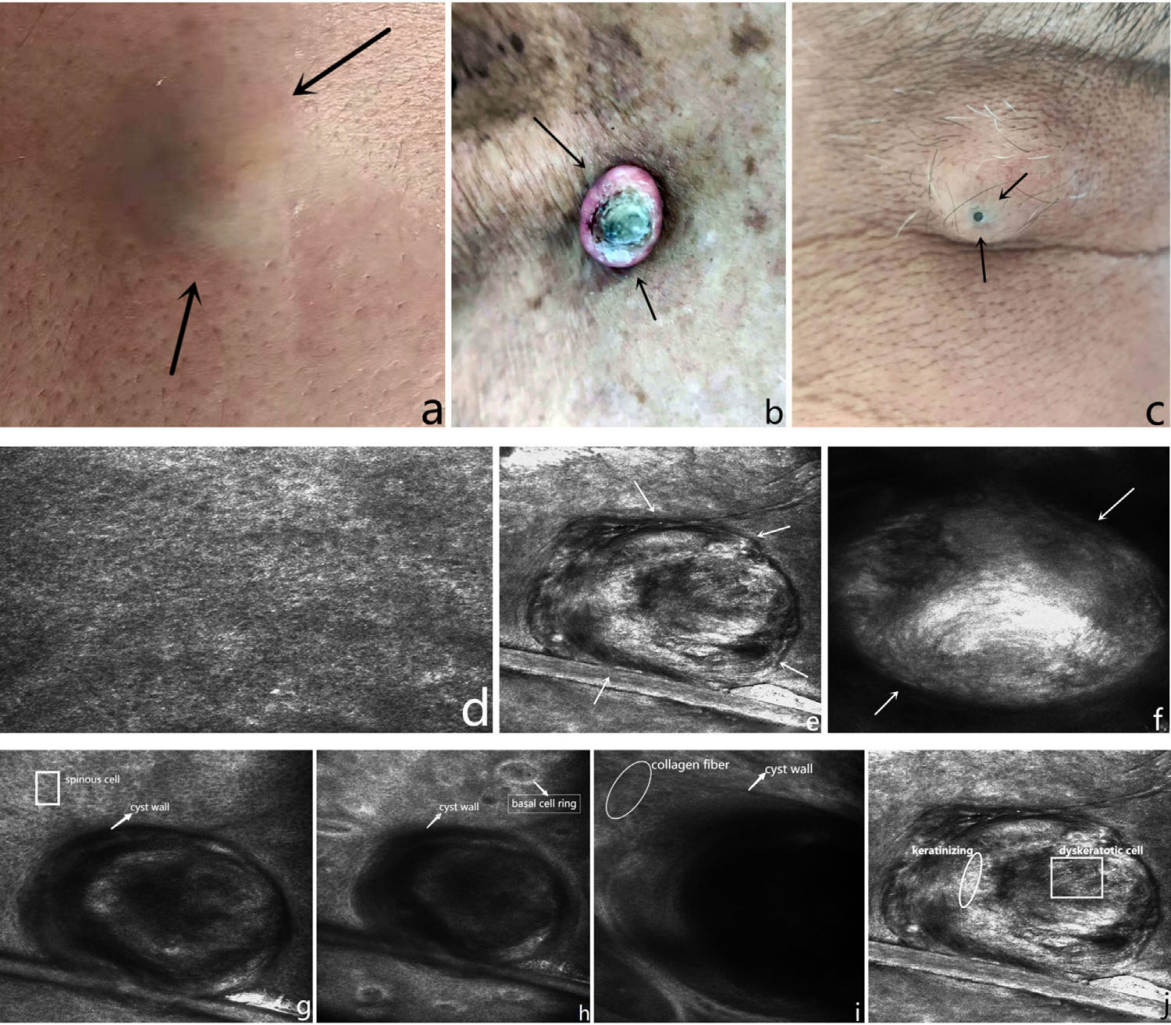
Clinical features and skin computed tomography (CT) image features of epidermoid cyst

Reflective confocal microscope is a new skin image diagnosis technology rising in recent years. It mainly obtains images through cross‐sectional continuous section scanning of skin lesions, which is mostly used for disease diagnosis and condition evaluation. At present, skin CT technology has been widely used in a variety of skin diseases. However, there is little exploration on the CT image features of epidermoid cyst. Epidermoid cyst, also known as dermal cyst,^1^ is one of the most common skin cysts. It often occurs on the face and upper trunk. The lesions are well‐defined nodules, and sometimes a central hole can be seen (Figure 1A–C). Small superficial epidermoid cysts are called miliary papules. Through the analysis of skin CT images of patients with epidermoid cyst, we found that the main skin CT features are as follows: (a) the epidermis is generally normal (Figure 1D); (b) high‐refraction cystic mass‐like circular structure with clear boundary can be seen in the superficial dermis, with uneven refraction (Figure 1E), and some skin CT manifestations similar to miliary papules (Figure 1F); (c) serial sections of skin lesions showed that the capsule wall was connected with the epidermis (Figure 1G–I); (d) the contents of the capsule are keratinized substances with medium and high refraction (Figure 1J); and (e) dyskeratotic cells with uneven refraction can sometimes be seen in the capsule cavity (Figure 1J).

**FIGURE 2:**
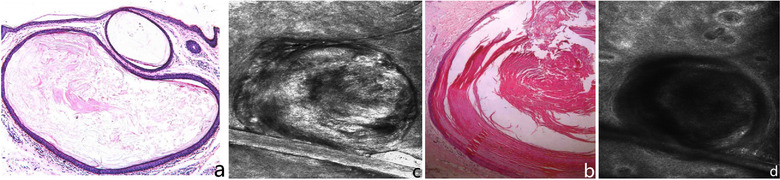
Connection between skin computed tomography (CT) image features of epidermoid cyst and its histopathological features

Compared with the typical histopathological features of epidermoid cyst, we found that there was a high consistency between its skin CT features and pathology. The typical histopathological features of epidermoid cyst are as follows: the cyst is located in the dermis, and the cyst wall is stratified squamous epithelium, which is similar to the infundibulum epithelium of hair follicle, that is, similar to the normal epidermis. From the outside to the inside, it is the basal cell layer, spinous layer, and granular layer. The contents of the cyst cavity are net basket or lamellar keratin, and sometimes some dyskeratotic cells can be seen. The capsule wall is connected with the epidermis. If one makes a continuous section, the part where the capsule wall is connected with the epidermis can be seen^2^ (Figure 2A,B). Through the above description, it is not difficult to find that many skin CT image features of epidermoid cyst have obvious correspondence with pathology, including lesion location, morphology, capsule contents, and capsule wall connected with epidermis (Figures 2C,D). These histopathological features can be reflected under skin CT. Therefore, to some extent, skin CT can replace histopathological biopsy to diagnose patients with clinically suspected epidermoid cyst.

Through the above information, we have preliminarily explored the skin CT image characteristics of epidermoid cyst, provided a new imaging diagnostic basis for the diagnosis of epidermoid cyst, and made the diagnosis more rapid, safe, and convenient, which will be beneficial to clinicians.
